# Device-measured capillary refill time identifies critically ill cases in the emergency department

**DOI:** 10.1038/s41598-025-34672-2

**Published:** 2026-01-12

**Authors:** Yayoi Miwa, Satoshi Karasawa, Takashi Shimazui, Takehiko Oami, Chiho Miyazawa, Masayoshi Shinozaki, Toshiya Nakaguchi, Taka-aki Nakada

**Affiliations:** 1 Department of Emergency and Critical Care Medicine, Chiba University Graduate School of Medicine, 1-8-1 Inohana, Chuo, Chiba, 260-8677 Japan; 2 Department of Medical Engineering, Graduate School of Science and Engineering, Chiba University, 1-33, Yayoicho, Inage, Chiba, 263-8522 Japan; 3 Augmented Living Technology Research Group (ALTec), Research Institute on Human and Societal Augmentation (RIHSA), National Institute of Advanced Industrial Science and Technology (AIST), University of Tokyo, Kashiwa II Campus, 6-2-3 Kashiwanoha, Kashiwa, Chiba 277-0882 Japan; 4 Center for Frontier Medical Engineering, Chiba University, 1-33Yayoicho, Inage, Chiba, 263-8522 Japan

**Keywords:** Capillary refill time, Emergency medical services, Lactic acid, Severity of illness index, Monitoring, physiologic., Medical research, Signs and symptoms

## Abstract

Capillary refill time (CRT) is a rapid, non-invasive indicator of tissue hypoperfusion and is associated with severity in critically ill patients. However, its clinical utility in the emergency department (ED) remains unclear, particularly when assessed using a quantitative device. We conducted a prospective, single-center observational study to evaluate whether device-measured CRT upon ED arrival is associated with clinical severity. One hundred nineteen adult patients transported to the ED by ambulance between August 2023 and October 2024 were enrolled. CRT was measured using the quantitative CRT device, and the average of three readings was analyzed. Critical illness was defined as an acute physiology and chronic health evaluation (APACHE) II score of ≥ 25. Among the participants, 27 (23%) had high APACHE II scores. CRT was significantly prolonged in this group (2.56 [1.84–3.36] vs. 1.67 [1.18–2.48] seconds, *p* = 0.0012) and showed a higher area under the receiver operating characteristic curve than lactate (AUC: CRT, 0.702; lactate, 0.669). CRT also demonstrated a weak but significant positive correlation with lactate levels (rs = 0.24, *p* = 0.0087) as well as a trend toward significant correlation with sequential organ failure assessment (SOFA) score (rs = 0.18, *p* = 0.051). These findings suggest that device-measured CRT is associated with clinical severity and may aid in the early identification of critically ill patients in emergency settings.

**Supplementary Information:**

The online version contains supplementary material available at 10.1038/s41598-025-34672-2.

**Original Article**.

## Introduction

Capillary refill time (CRT) is a valuable indicator of tissue hypoperfusion in critically ill patients. Therefore, it has been used to assess the severity of critical illness^1–5^. Early identification of tissue hypoperfusion is crucial for timely detection of organ dysfunction. Lactate is a biomarker commonly used to assess tissue hypoperfusion; however, CRT offers the advantage of being a simple, non-invasive alternative that does not require blood sampling. Consequently, CRT measurement is beneficial for the early identification of critically ill patients, which is a critical step in their clinical management.

Prolonged CRT is associated with increased mortality in critically ill patients, including those with septic and cardiogenic shock^4,6^. Given the simplicity and rapid applicability of CRT, its use in the early stage of patient care such as emergency department (ED) may be advantageous. However, the number of studies on this topic is limited. Severity is typically assessed based on vital signs upon arrival at the ED. Owing to its ease of measurement, the incorporation of CRT may improve the accuracy of early severity prediction without delaying management.

Traditionally, CRT is assessed by applying pressure to the nail bed and visually observing the return of color. However, because this method is prone to inter-observer variability, it is potentially inaccurate^7^. The use of quantitative measurement devices allows for a more precise CRT assessment, and recent investigations have reported that device-based CRT measurements have contributed to the accurate diagnosis of sepsis in the ED^8^. We developed a quantitative CRT device (qCRT device) capable of quantitatively assessing CRT^8^. Although the agreement between traditional visually measured CRT and device-measured CRT is weak due to differences in measurement mechanisms, and the absolute CRT values should therefore be interpreted with caution, the device-measured CRT has been reported to be useful as it reduces interobserver variability compared with visual measurement and enables objective, stable, and reliable assessments^8^. The application of this device for CRT measurements in ED patients may aid in early severity prediction. However, no study has evaluated CRT using such a quantitative device across ED patients without limiting the evaluation to specific disease categories.

In this study, we hypothesized that CRT measured using a quantitative device could predict the severity of symptoms in patients presenting to the ED. To test this hypothesis, we prospectively evaluated the CRT and assessed its association with other indicators of severity.

## Methods

### Study setting and patients

**Fig. 1:**
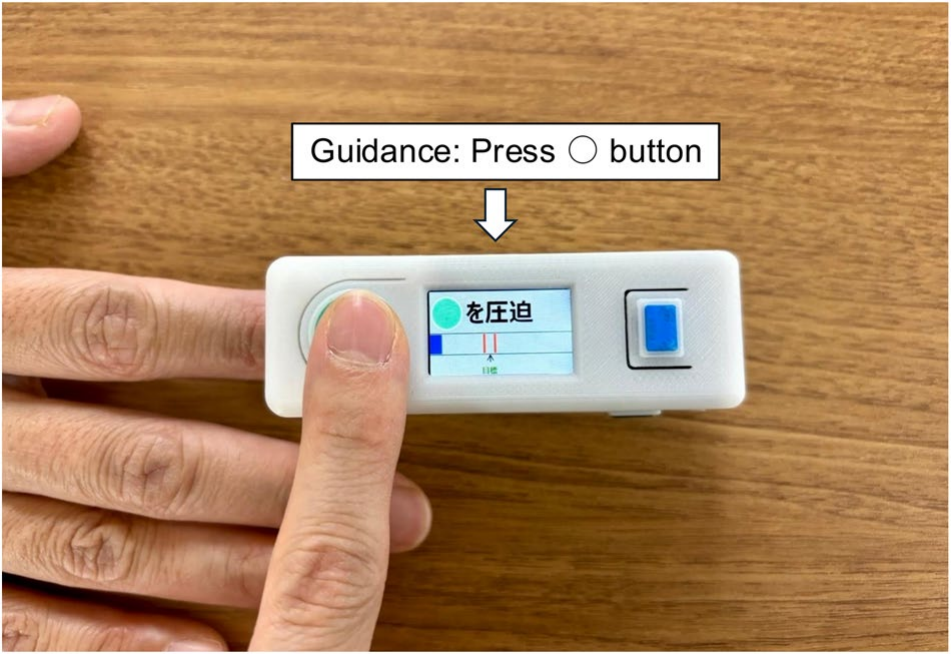
Quantitative capillary refill time device. Portable device for the quantitative measurement of capillary refill time. It guides the user in applying standardized pressure to the nail bed through visual prompts displayed on the screen.

This single-center prospective observational study was conducted at Chiba University Hospital, which accommodates approximately 5,700 emergency department visits annually. The study enrolled adult patients (≥ 18 years) who presented to the ED by ambulance and were treated by emergency physicians between August 2023 and October 2024, excluding those in cardiac arrest. CRT was measured upon ED presentation using the qCRT device (Fig. 1)^8^ by researchers or research assistants who had been trained in its use. Although the number of trained staff was limited, patient enrollment was not restricted by illness severity or diagnosis, and CRT measurements were attempted as broadly as operationally feasible (Supplementary Fig. S1). The Chiba University Hospital Ethics Committee for Observational Studies approved this study (approval number: M10800). Written informed consent was obtained from all study participants and/or their representatives. Because this was an observational study, trial registration was not required and was therefore not performed. All methods were performed in accordance with the relevant guidelines and regulations.

### Data collection and definition

The following data were prospectively collected from the enrolled patients: age, sex, height, weight, vital signs (systolic blood pressure [SBP], diastolic blood pressure, heart rate [HR], respiratory rate, Glasgow Coma Scale [GCS], and body temperature), diagnosis at the time of hospital admission, blood lactate levels, sequential organ failure assessment (SOFA) score, hospitalization status, in-hospital mortality, and acute physiology and chronic health evaluation (APACHE) II score. APACHE II scores were calculated based on the worst values recorded within 24 h after admission; for patients who were not admitted, based on the worst values in the emergency department. The diagnoses were categorized as trauma, infection, cardiovascular disease, or other diseases.

CRT was measured using the qCRT device capable of quantitatively assessing CRT by analyzing changes in the nail bed color tone using reflected light^8,9^ (details of the measurement principle and methodology are provided in Supplementary Materials, including Supplementary Fig. S2 and S3). The average of three consecutive measurements was used for the analysis. Waveform analysis was performed to evaluate the rate of color recovery following the release of pressure on the nail bed. Measurements that exhibited abnormal waveforms, indicating an insufficient recovery rate, were excluded from the analysis^10^.

The APACHE II score was evaluated as an indicator of critical illness because it is widely used in critically ill patients and strongly associated with mortality^11^. To classify patients as critically ill or not, study participants were categorized into two groups; the High APACHE II group (APACHE II score ≥ 25) or Low APACHE II group (< 25) based on previous reports^12–14^.

Lactate is a biomarker that is widely recognized as reflecting disease severity^15–19^. Thus, we also analyzed the association between CRT and lactate levels. Patients were stratified into two groups based on their lactate levels; the High lactate group (> 2.0 mmol/L) and the Low lactate group (≤ 2.0 mmol/L), based on previous reports^20^.

### Statistical analysis

The primary analysis examined the association between CRT and critical illness, defined as an APACHE II score of ≥ 25, as CRT is traditionally associated with severity^3,5^. The analytical approach to assess the association with the severity score was defined prior to conducting this prospective study. Secondary analyses included the associations between CRT and lactate levels as well as in-hospital mortality rates, and the correlation with SOFA score. CRT was also compared between the patients who survived and those who did not. Logistic regression analyses were conducted to further evaluate the independent association between CRT and critical illness. Baseline covariates such as age and sex were adjusted based on the previous reports^21,22^. A priori sample size calculation could not be performed due to the lack of established data on the effect of device-measured CRT values in critically ill patients presenting to the ED. Therefore, a post hoc power analysis was conducted based on the observed CRT values, the actual sample size, and a two-sided α of 0.05, which yielded an estimated statistical power of approximately 0.88.

The Mann–Whitney U test or Fisher’s exact test was used to evaluate differences between two groups, and the Kruskal–Wallis test was used to evaluate differences among multiple groups. Dunn’s multiple comparisons test was used for post hoc comparisons between Q4 and each of the other quartiles, since Q4 showed different CRT values compared with the other groups. The correlation of CRT with outcomes was evaluated using Spearman’s rank correlation coefficient. The discriminatory performance of CRT in identifying critical illness was assessed by calculating the area under the receiver operating characteristic (ROC) curve (AUC). The AUCs of other commonly available indicators that have been reported to be associated with severity in the ED, such as lactate, mean arterial pressure (MAP), systolic blood pressure (SBP), and heart rate (HR)^23–25^, were also evaluated. A multivariable model was used to calculate the AUCs for combinations of these factors. The optimal cutoff value was determined using the Youden index, and its sensitivity and specificity were calculated. Continuous variables are presented as medians (interquartile ranges [IQR]), and categorical variables are presented as numbers (%). Statistical significance was defined as a two-sided P value < 0.05. Statistical analyses were performed using JMP Pro 18 (SAS Institute Inc., Cary, NC, USA), GraphPad Prism 10 (GraphPad Software, San Diego, CA, USA), R version 4.4.3 (R Foundation for Statistical Computing, Vienna, Austria), and G*Power 3.1 (Heinrich Heine University Düsseldorf, Düsseldorf, Germany).

## Results

A total of 119 patients were included in the study. The APACHE II score across all patients was 14 [IQR 8–24], and the SOFA score was 2 [IQR 0–17]. Twenty-seven patients (23%) were classified into the High APACHE II group and 92 patients (77%) into the Low APACHE II group.

The High APACHE II group was significantly older and had a lower incidence of trauma and a higher incidence of infections (Table 1). Additionally, the High APACHE II group exhibited significantly lower systolic and diastolic blood pressure and GCS, and elevated lactate levels. Moreover, the High APACHE II score group had higher rates of hospitalization and in-hospital mortality (Table 1).

**Table 1:** Baseline characteristics and clinical outcomes of the two groups stratified by APACHE II score.

	Low APACHE II *n* = 92	High APACHE II *n* = 27	*P* value
Characteristic			
Age, years	63 (45–79)	79 (68–85)	0.0010
Male sex, n (%)	34 (37.0)	8 (29.6)	0.64
Lactate (mmol/L)	1.8 (1.2–3.2)	4.1 (1.6–8.6)	0.0076
Height (cm)	164 (155–171)	159 (138–167)	0.13
Weight (kg)	61 (49–72)	54 (46–64)	0.10
Vital signs			
Systolic blood pressure (mmHg)	135 (115–156)	115 (90–142)	0.028
Diastolic blood pressure (mmHg)	83 (69–97)	71 (64–85)	0.020
Heart rate (/min)	89 (75–107)	102 (70–122)	0.42
Respiration rate (/min)	20 (17–24)	21 (12–30)	0.36
Glasgow coma scale	15 (14–15)	10 (5–15)	< 0.0001
Body temperature (ºC)	36.6 (36.3–37.2)	36.8 (36.4–37.6)	0.26
Diagnosis on admission - n (%)			
Trauma	37 (40.2)	2 (7.4)	0.0010
Infection	8 (8.7)	9 (33.3)	0.0032
Cardiovascular disease	5 (5.4)	5 (18.5)	0.046
Others	42 (45.6)	11 (40.7)	0.99
Outcome			
Hospital admission - n (%)	66 (71.7)	26 (96.3)	0.0076
In-hospital mortality - n (%)	3 (3.3)	5 (18.5)	0.015

Data are presented as medians and interquartile ranges for continuous variables and exact numbers (%) for categorical variables. P values were calculated using the Mann–Whitney U test or Fisher’s exact test.

APACHE, acute physiology and chronic health evaluation.

### Comparison of capillary refill time by APACHE II score classification

**Fig. 2:**
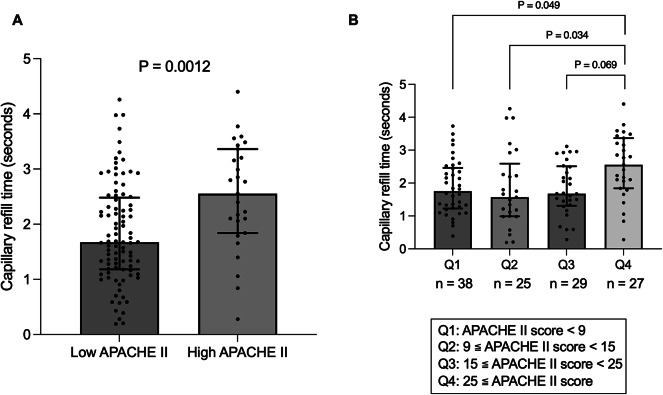
Comparison of capillary refill time in classification by APACHE II score. (**A**) Classification by APACHE II score cutoff of 25. Capillary refill time was significantly prolonged in the High APACHE II group compared to that in the Low APACHE II group. (*P* = 0.0012). (**B**) Classification into four groups according to APACHE II score quartiles. Capillary refill time was longest in Q4 (APACHE II ≥ 25) and differed significantly among the groups (*P* = 0.016). Dunn’s multiple comparison post hoc tests showed significant differences between Q4 and Q1 or Q2 (Q1 vs. Q4, *P* = 0.049; Q2 vs. Q4, *P* = 0.034), while Q3 showed a non-significant trend (*P* = 0.069). APACHE, acute physiology and chronic health evaluation.

CRT was significantly prolonged in the High APACHE II group compared to that in the Low APACHE II group (2.56 [1.84–3.36] vs. 1.67 [1.18–2.48] seconds, *P* = 0.0012) (Fig. 2A). When patients were stratified into four groups based on APACHE II score quartiles (APACHE II score, Q1 < 9, 9 ≤ Q2 < 15, 15 ≤ Q3 < 25, 25 ≤ Q4), CRT values significantly differed among the groups (*P* = 0.016). Notably, patients in the fourth quartile (Q4), corresponding to an APACHE II score of ≥ 25 (the same threshold used to define the High APACHE II group) had the longest CRT values among all groups (*P* = 0.016). Dunn’s multiple comparison post hoc analysis showed significant differences between Q4 and Q1 or Q2 (Q1 vs. Q4, *P* = 0.049; Q2 vs. Q4, *P* = 0.034), while Q3 showed a non-significant trend (*P* = 0.069) (Fig. 2B).

### Capillary refill time as a predictor of high APACHE II score

Univariate and multivariable logistic regression analyses showed that higher CRT was significantly associated with a high APACHE II score, indicating that CRT is an independent predictor of critical illness even after adjusting for background variables (univariate: OR 2.12; 95% confidence interval [CI] 1.33–3.53; *P* = 0.0022; multivariable; adjusted OR 1.86; 95% CI 1.16–3.12; *P* = 0.013) (Supplementary Table 1).

### Discriminatory performance of capillary refill time and other circulatory indicators

**Fig. 3:**
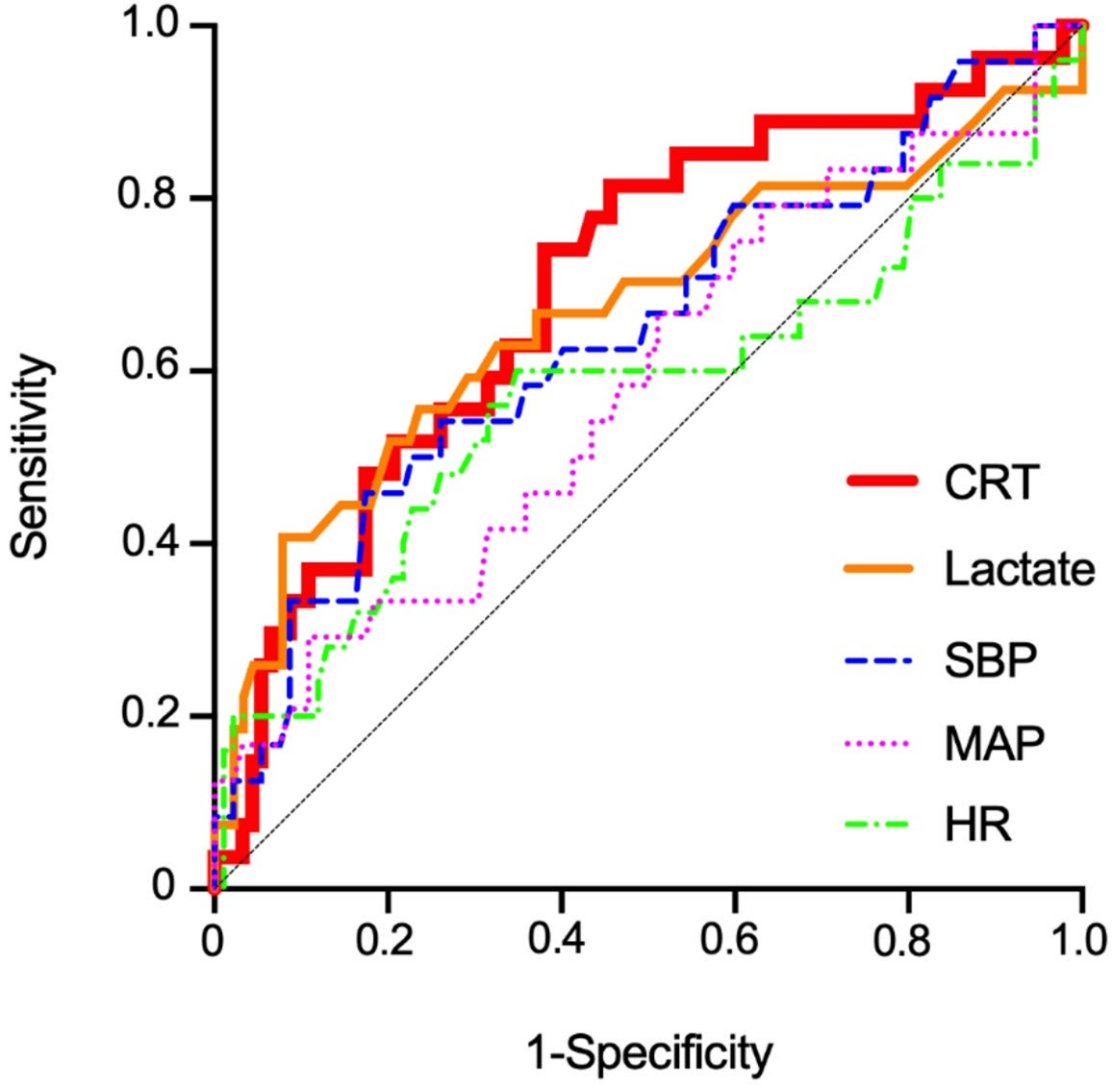
Receiver operating characteristic curve analysis for discriminating High APACHE II patients using circulatory indicators. Capillary refill time demonstrated the highest area under the curve compared to other indicators. APACHE, acute physiology and chronic health evaluation; CRT, capillary refill time; SBP, systolic blood pressure; MAP, mean arterial pressure; HR, heart rate.

In the analysis of the performance in identifying high APACHE II scores, CRT demonstrated the highest AUC among all evaluated variables (AUCs, CRT, 0.702; Lactate, 0.669; SBP, 0.645; MAP, 0.586; HR, 0.574), with a cutoff value of 2.05 s (Table 2; Fig. 3). Combining CRT and lactate levels increased the AUC to 0.740. Upon addition of MAP to the model, the AUC increased further to 0.753.

**Table 2:** Receiver operating characteristic curve analysis for identifying high APACHE II patients using circulatory indicators.

Variable	AUC (95% CI)	Sensitivity	Specificity	Cutoff value
CRT	0.702 (0.587–0.817)	0.740	0.620	2.05 s
Lactate	0.669 (0.536–0.801)	0.407	0.921	5.65 mmol/L
SBP	0.645 (0.513–0.777)	0.458	0.826	109.5 mmHg
MAP	0.586 (0.451–0.720)	0.750	0.554	100.0 mmHg
HR	0.574 (0.428–0.720)	0.577	0.652	96.0/min

The Youden index was used to determine the cutoff values.

APACHE, acute physiology and chronic health evaluation; CRT, capillary refill time; SBP, systolic blood pressure; MAP, mean arterial pressure; HR, heart rate; AUC, area under the curve; CI, confidence interval.

### Association of capillary refill time with lactate levels and SOFA score

**Fig. 4:**
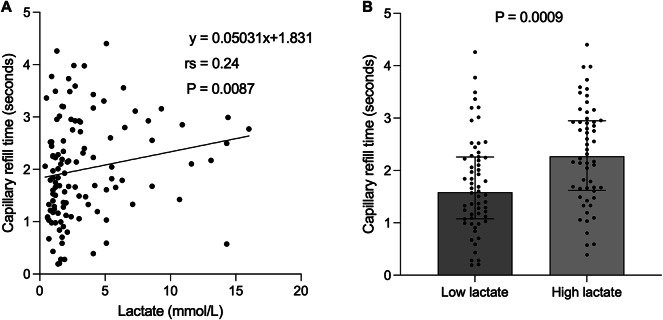
Association between lactate and capillary refill time. (**A**) Correlation between lactate and capillary refill time. Spearman’s rank correlation revealed a significant weak positive correlation (rs = 0.24, *P* = 0.0087). (** B**) Comparison of capillary refill time in classification by lactates. CRT was significantly prolonged in the high lactate group compared to that in the low lactate group. (*P* = 0.0009).

Lactate levels showed a significantly weak positive correlation with CRT (rs = 0.24, *P* = 0.0087) (Fig. 4A). When CRT was compared between the High and Low lactate groups (*n* = 56 vs. 60; missing lactate level, *n* = 3), CRT was significantly prolonged in the High lactate group (2.27 [1.62–2.94] vs. 1.59 [1.08–2.26] seconds, *P* = 0.0009) (Fig. 4B). The SOFA score also showed a trend toward a significant correlation with CRT (rs = 0.18, *P* = 0.051) (Fig. S4).

### Association between capillary refill time and in-hospital mortality

There was no statistically significant difference in CRT between the in-hospital survivors and the non-survivors (*n* = 111 vs. 8) (1.92 [1.33–2.72] vs. 2.66 [1.30–3.43] seconds, *P* = 0.22).

## Discussion

CRT was significantly prolonged in the High APACHE II score group, indicating its association with critical illness in patients who presented at the ED. Furthermore, higher CRT is independently associated with a high APACHE II score and demonstrated the highest AUC for predicting High APACHE II scores. A weak correlation was observed between CRT and lactate levels as well as SOFA score, with CRT being significantly elevated in the High lactate group compared to that in the Low lactate group. No statistically significant difference in CRT was observed between the survivors and non-survivors.

CRT is associated with critical illnesses in patients presenting to the ED. A previous study involving 282 ICU patients reported that prolonged CRT correlated with impaired microcirculatory parameters, such as microvascular flow index, proportion of perfused vessels, heterogeneity index, and perfused vessel density, as measured by sublingual microcirculation imaging. It is also associated with higher SOFA scores and increased mortality^3^. A meta-analysis of critically ill patients in both the ED and ICU reported a significant association between prolonged CRT and increased three-month mortality^6^. Consistent with these reports, our study demonstrates a significant association between CRT measured using the qCRT device and critical illness assessed using the APACHE II score in the ED. While there was no statistically significant difference between Q3 and Q4 (*P* = 0.069), and caution is required when interpreting whether CRT can distinguish higher APACHE II scores, the post hoc analysis across APACHE II score quartiles showed that the highest quartile (Q4) had the longest CRT and that CRT differed significantly in comparisons involving Q1 and Q2. One possible explanation for the failure to observe an association between CRT and mortality is the limited number of deaths (*n* = 8), which may have resulted in insufficient statistical power to detect such an association. Further studies with larger sample sizes are warranted.

As a possible mechanistic explanation for CRT’s association between CRT and critical illness, a pilot study of 30 patients with septic shock demonstrated a positive correlation between CRT and intestinal pulsatility indices measured using Doppler ultrasound (Pearson’s *r* = 0.325, *P* = 0.007), which reflects the vascular tone in the intestines^26^. This phenomenon is thought to result from compensatory vasoconstriction during shock, which redistributes blood flow from peripheral tissues such as the skin and intestines to vital organs including the heart, lungs, and brain. CRT may reflect visceral ischemia, which is closely associated with organ dysfunction and disease severity. This finding supports its discriminatory value in identifying critically ill patients.

CRT and lactate are both established indicators of tissue hypoperfusion^27,28^. In our study, CRT showed a weak correlation with lactate levels. Additionally, CRT had a higher AUC for critical illness than lactate levels. Lactate levels, although indicative of tissue hypoperfusion, can also be elevated because of other causes such as seizures, hematologic malignancies, or hepatic dysfunction^28^. In terms of the ability to discriminate severity, CRT exhibited a higher AUC than lactate, suggesting that CRT may reflect circulatory insufficiency and acute physiological deterioration more specifically. CRT also has the advantage of being a convenient, noninvasive, and rapid assessment tool, making it well suited for application in prehospital settings. Furthermore, combining CRT with other parameters such as lactate and MAP may increase discriminatory performance, as indicated by the AUC values. This integrative approach may facilitate the earlier identification and treatment of critically ill patients, potentially improving clinical practice.

Although numerous studies have investigated CRT, few have focused on quantitative measurements using dedicated devices. Visual assessments are prone to inter-observer variability^7^, highlighting the need for standardized evaluation. Our qCRT device provides quantitative values with greater stability and less variability compared to visually assessed CRT^8^. The clinical utility of other physiological measurements such as automated pupillary assessments has recently gained attention^29^, underscoring the broad relevance of objective monitoring tools. While previous studies have reported CRT cutoff values ranging from 2 to 4 seconds^6,30^, our previous study in healthy adults found a median CRT of 2.88 seconds^10^, and the present study identified a prediction cutoff of 2.05 s. Another study utilizing a CRT device based on a similar measurement principle reported a sepsis screening threshold of 3.5 seconds^31^. These differences suggest that optimal cutoff values may vary depending on the clinical context and population, warranting further investigation to establish generalized thresholds. Considering the measurement principles and based on our previous report^8^, agreement between the device-measured and visually assessed CRT may not be observed. In addition, the visual detection of CRT differences of less than one second is inherently challenging, reinforcing the importance of quantitative assessments using devices.

A major strength of this study is its comprehensive evaluation of CRT in ED patients across a broad clinical spectrum using a quantitative device. However, this study had several limitations. First, this was a single-center study with a relatively small sample size. Additionally, the sample size calculation was performed post hoc using the observed effect size, which limits the ability to formally assess whether the study was adequately powered to detect clinically meaningful differences and may over- or underestimate the true effect, rather than being based on prespecified hypotheses. However, although the small number of trained researchers available to perform CRT measurements limited the sample size, the data were collected from patients with a broad range of etiology and severities. Second, CRT can be influenced by factors such as race, ambient temperature, and lighting conditions^32,33^; however, data on these variables were not collected in this study. Third, CRT was measured at a single time point upon ED admission, and longitudinal changes over time were not assessed. To fully evaluate the prognostic value of quantitative CRT measurements, future studies should examine temporal trends and their associations with clinical outcomes, including mortality, in larger patient cohorts. Fourth, although the device used in this study was designed to reduce the subjectivity and variability of visual CRT assessments^8^, visual CRT was not recorded simultaneously. We have previously reported that the agreement between device-measured and visually assessed CRT is weak because of differences in their measurement principles^8,9^. Therefore, CRT derived from the device evaluated in this study and manually measured CRT may represent different parameters and may not have the same clinical value. A direct comparison might have highlighted the superiority of device measurement; however, we were unable to compare the diagnostic utility for outcomes. Fifth, this study did not include analyses of other severity scores, such as the NEWS2 score. Data collection for the components of these scores was not pre-scheduled, and because the data was not obtained at appropriate times, their assessment would not have been accurate. Further studies are needed to evaluate the relevance of quantitative CRT for other outcomes and to assess its utility from multiple perspectives.

## Conclusion

This study determined that device-measured CRT was associated with critical illness in patients who presented at the ED. In addition, CRT is associated with lactate levels and has a higher discriminatory value for critical illnesses. The early identification of critically ill patients through CRT assessment has the potential to enhance the timeliness of care delivery and improve the overall quality of emergency care.

## Supplementary Information

Below is the link to the electronic supplementary material.

Supplementary Material 1

## Data Availability

The datasets generated and analyzed during the current study are available from the corresponding author upon reasonable request.
