# Supplementary material for: Device-measured capillary refill time identifies critically ill cases in the emergency department
Source: Sci Rep. 2026 Jan 12;16:4620. doi: 10.1038/s41598-025-34672-2 (PMC12867985; doi:10.1038/s41598-025-34672-2)
Supplement: Supplementary file 1 — Supplementary Material 1 [file 41598_2025_34672_MOESM1_ESM.docx]

**Device-measured capillary refill time identifies critically ill cases in the emergency department**

Yayoi Miwa, Satoshi Karasawa, Takashi Shimazui, Takehiko Oami, Chiho Miyazawa, Masayoshi Shinozaki, Toshiya Nakaguchi, Taka-aki Nakada

Supplementary Materials

Supplementary methods

In this study, we utilized a compact device (2.8 cm × 8.5 cm × 3.1 cm) developed in-house to enable quantitative measurement of capillary refill time (CRT) ^1,2^ (Supplementary Fig. S2). As previously described ^3,4^, the device emits white light onto the fingertip and quantifies the CRT by analyzing variations in the green component of the reflected light. Because green wavelength light is readily absorbed by the blood, it exhibits greater sensitivity to changes in peripheral blood flow, thereby enhancing the signal-to-noise ratio. The color of the nail bed under compression was defined as 100%, and the stabilized color after release was defined as 0%. CRT was calculated as the time required for the color to transition from 90% to 10% ^5^.

The force and duration of the nail bed compression were standardized using a feedback mechanism integrated into the device. This standardization effectively eliminates inter-operator variability in the applied pressure and compression duration, resulting in more consistent and reliable CRT measurements ^1,6^ (Supplementary Fig. S3). Each measurement could be completed within several tens of seconds, allowing three repetitions per patient to be performed within a few minutes. In accordance with previous studies [2], CRT in this study was measured with patients in the supine position and their right upper extremity positioned at heart level. The measurement was taken on the patients’ right index finger.

**Supplementary Figure S1. Flow chart of the study**

CRT, capillary refill time; APACHE, acute physiology and chronic health evaluation.

**Supplementary Figure S2. Quantitative capillary refill time device**

A. Device components: (1) compression point, (2) display, (3) reset switch, and (4) power switch.

B. Device component: (5) force and color sensors, where the examinee's finger is placed.

**Supplementary Figure S3. Measurement process**

Quantitative capillary refill time device including feedback system of measuring conditions. The measurer follows the indications on the display and compresses the patient's nail bed to gather the data under the optimal conditions for the capillary refill time measurement.

**Supplementary Figure S4. Correlation between SOFA score and capillary refill time**

Spearman’s rank correlation showed no significant correlation between SOFA score and CRT, although a trend toward a positive correlation was observed (rs = 0.18, P = 0.051).

SOFA, sequential organ failure assessment.

**Supplementary Table 1. Logistic regression analysis of the identification of factors predictive of high APACHE II score**

**A. Univariate**

|  | **Odds ratio (95% CI)** | **P-value** |
| --- | --- | --- |
| Capillary refill time | 2.12 (1.33–3.53) | 0.0022 |

**B. Multivariable**

|  | **Adjusted odds ratio (95% CI)** | **P-value** |
| --- | --- | --- |
| Capillary refill time | 1.86 (1.16–3.12) | 0.013 |
| Age | 1.04 (1.01–1.08) | 0.0048 |
| Male sex | 0.94 (0.71–2.00) | 0.53 |

APACHE, acute physiology and chronic health evaluation; CI, confidence interval.

**References**

[1] Shinozaki, M. et al. Feedback function for capillary refilling time measurement device. *Critical Care.* **23** (2019).

[2] Saito, D. et al. Impact of posture on capillary refilling time. *The American Journal of Emergency Medicine.* **56**, 378-379 (2022).

[3] Shinozaki, M., Shimizu, R., Saito, D., Nakada, T.-a. & Nakaguchi, T. Portable measurement device to quantitatively measure capillary refilling time. *Artificial Life and Robotics.* **27**, 48-57 <https://doi.org/10.1007/s10015-021-00723-w> (2022).

[4] Bachour, R. P. S., Dias, E. L. & Cardoso, G. C. Skin-color-independent robust assessment of capillary refill time. *J Biophotonics.* **16**, e202300063 (2023).

[5] Kawaguchi, R. et al. Optimal pressing strength and time for capillary refilling time. *Critical Care.* **23** (2019).

[6] Sheridan, D. C., Cloutier, R. L., Samatham, R. & Hansen, M. L. Point-Of-Care Capillary Refill Technology Improves Accuracy of Peripheral Perfusion Assessment. *Front Med (Lausanne).* **8**, 694241 <https://doi.org/10.3389/fmed.2021.694241> (2021).
